# Acceptability of a Self-Help Programme to Address the Use of Indecent Images of Children

**DOI:** 10.5964/sotrap.11159

**Published:** 2024-12-03

**Authors:** Sarah Wefers, Alexandra Bailey, Nadia Rasooli, Donald Findlater, Lucy Allen

**Affiliations:** 1 Lucy Faithfull Foundation, Epsom, United Kingdom; 2 College of Biomedical and Life Sciences, School of Medicine, Cardiff University, Cardiff, United Kingdom; 3 School of Psychology, University of Roehampton, London, United Kingdom; University Medical Center Mainz, Mainz, Germany

**Keywords:** prevention, self-help, indecent images of children, child sexual abuse material, evaluation, acceptability

## Abstract

The use of indecent images of children (IIOC) is of continued concern and growing prevalence. A multi-agency approach to this online crime is necessary, as it cannot be eradicated by law enforcement alone. Previous research has examined the pathways to offending for this population, and prevention strategies that could be used to deter offending in the first instance, or to stop behaviour once it has begun. The current qualitative study aimed to explore acceptability of a prevention initiative; a self-help focussed service (a website and calls with a professional). Semi-structured interviews with eight individuals who had accessed IIOC and engaged in this self-help focussed service were conducted. Transcripts were analysed using qualitative framework approach, using the Theoretical Framework of Acceptability (TFA) domains as deductive codes. All seven domains of the TFA were identified in the transcripts, indicating the applicability of the framework. Overall, participants reported mixed acceptability of and satisfaction with the self-help programme and calls with practitioners, but high perceived effectiveness in the service supporting them stop their illegal behaviours. The implications of the study are considered in line with improvements that could be made to this existing self-help prevention initiative.

## Abstract (plain language summary)

**Background:**

The use of indecent images of children (IIOC) online causes severe harm to children and society. The high numbers of offences including such material cannot be tackled by law enforcement alone and needs prevention and intervention, helping people who have offended or are at risk of offending to stop.

**Why was this study done?:**

Some self-help programmes for people at risk of online offending exist, but it is not yet known how well they work. This study explored how acceptable users of one self-help programme called Get Help found the programme to help understand how such programmes should be designed and delivered.

**What did the researchers do and find?:**

Users of the Get Help programme were interviewed about their experience of using the self-help material and discussing them with a practitioner. Feedback included that some participants found parts of the material difficult to work through, due to the negative emotions it brought up, the website being difficult to navigate, or due to additional needs like dyslexia. Participants reported that the modules helped them in stopping and continuing their desistance from the illegal behaviour.

**What do these findings mean?:**

Self-help programmes can help people in not engaging in illegal online behaviours. Websites that host such self-help programmes should be easily accessible, anonymous to use, and professional support should be offered for users to help them work through the material.

## Highlights

- Online self-help programmes provide low-threshold support for people who are concerned about their use of indecent images of children.
- Online self-help programmes can help users of indecent images of children to stop and continue desisting from their illegal behaviours.
- Support from practitioners may improve outcomes for users of such online self-help programmes.

The challenge of targeting the use of indecent images of children (IIOC) online is not new, but an ever-growing problem in today’s technological world (Westlake, 2019). The growing presence, access and distribution of these images (see e.g. Bryce et al., 2023) contribute to the continued sexual abuse and exploitation of large numbers of children around the world; for those victimised within the images, the continued circulation of their pictures has been shown to exacerbate feelings of shame (Gewirtz-Meydan et al., 2018).

The prevalence of individuals accessing IIOC has continued to increase over time. In the UK, arrests for those accessing IIOC were at around 500 per month in 2020 (Mee, 2020). The National Society for the Prevention of Cruelty towards Children (NSPCC) reported that more than 100,000 IIOC offences were recorded in the UK between 2016 and 2021, with a peak of recorded offences in 2020/2021 (NSPCC, 2021). Similar trends have been noted in other European countries. For example, the Federal German Police (2022) reported an increase of 108% from 2020 to 2021 in child abuse material offences and EUROPOL (2020) noted an increase in online child sexual offences, particularly during COVID-19 lockdowns. However, despite an increased police response to IIOC offending, it is widely recognised that tertiary prevention strategies, such as arrest and conviction, are not sufficient to address this rising issue (Sawer, 2017).

For that reason, increasing emphasis is being placed on prevention initiatives to deter individuals from accessing IIOC in the first place, or to encourage those already accessing to cease their online behaviour. Attention has been focussed on primary and secondary prevention, with the use of a public health model of child sexual abuse prevention being argued by those within the field (Brown et al., 2012; McMahon & Puettl, 1999). The Comprehensive Framework for the Prevention of Child Sexual Abuse (Smallbone et al., 2008) provides a structure by which to consider different levels of prevention for child safeguarding, influenced by situational crime prevention and Routine Activity Theory (Cohen & Felson, 1979). In essence it is underpinned by the premise that an offence can occur when there is an appropriate target or potential victim, a person motivated to offend, and the lack of presence of a guardian. This indicates that certain situations can facilitate offending behaviour. It follows that if a person’s situation can facilitate offending, addressing that same situation could be used in attempts to prevent the offending behaviour from occurring. This can include both changing the environment itself, or by using the situation to influence the decision-making process of someone at risk of offending, such as by increasing risk, increasing effort, removing excuses/reducing permissibility and using prompts to deter offending behaviour (Wortley & Smallbone, 2012).

Situational crime prevention frameworks may also be applied to the availability of and access to IIOC. The Internet Watch Foundation (IWF) removes IIOC from the online space and thereby makes it less likely that individuals may reach a situation in which they can offend. The IWF removed images and videos from over 132,000 webpages in 2019 (IWF, 2020) and has actioned 970,000 reports of IIOC for removal between 1996 and 2021 (IWF, n.d.). The IWF work to ‘increase the effort’ required to access this material (Wortley & Smallbone, 2012). Initiatives from search engines such as Google continue this work by failing to provide search results for illegal material, with online warning pages and other adverts aiming to ‘remove excuses’ by informing those looking for IIOC that this is illegal (Wortley & Smallbone, 2012). Prichard and colleagues (2022) showed that online warning messages are effective in deterring people from accessing risky sexual material online, and that particularly messages about the illegality of material are effective. This demonstrates the use of situational crime prevention and the need to identify the most effective messaging for deterrence efforts.

The largest national secondary child sexual abuse prevention initiative in the UK to date is the deterrence campaign run by the Lucy Faithfull Foundation. The Lucy Faithfull Foundation is a child protection charity focused on the prevention of child sexual abuse. The aim of the deterrence campaign is to encourage those who are offending, or at risk of offending, online to cease the behaviour and seek help. This is achieved via online deterrence messaging providing information about the consequences of online offending, as well as information about accessing anonymous help. Bailey and colleagues (2022) conducted qualitative interviews with 20 individuals who had come to police attention for accessing IIOC. Participants’ pathways to the offending behaviour were explored, as well as what participants believed may have prevented their offending, or resulted in desistance. The authors identified key themes relating to how prevention initiatives could prevent the initial access of IIOC, or encourage desistence once offending had begun. This included targeting participants’ thoughts about their offending behaviour, such as a perceived lack of harm caused, and a naivety of the law and of the consequences of their behaviour. Similar thinking styles, such as the minimisation and justification of offending behaviour, have previously been identified as cognitive distortions or broader offence-supportive cognitions acting as facilitators to IIOC users’ online behaviours (Kettleborough et al., 2016). In addition, participants identified a perceived lack of professional support and subsequently, the importance of available confidential help for those who have concerns about their online behaviours was highlighted.

As a result of the research by Bailey et al. (2022), a UK-wide media deterrence campaign was developed in an attempt to address the areas above. One section of the campaign focused on the distribution of public messages within online environments. These messages focused on the illegality and consequences of accessing IIOC, and encouraging those concerned about their online behaviour to seek help. Alongside this campaign was the redesign of an anonymous online self-help resource for those concerned about viewing IIOC, called Get Help. The website provided information and online modules, with the aim of aiding individuals in understanding their online sexual behaviour, and implementing management strategies. Bailey et al. (2022) highlighted the importance of a website like Get Help, where individuals could receive accurate and up to date information about the law in the UK and the consequences of viewing IIOC, but could also obtain help in a way that did not compromise their anonymity. A number of significant changes were made to the website, including updating the self-help information with more exercises to encourage continued engagement. The website additionally created a strength-based focus based on The Good Lives Model (Ward, 2002), encouraging individuals to consider positive goal-oriented change, rather than the relapse prevention focus of the original Get Help site.

To support users in working through the online self-help modules, users could engage in a series of calls with a practitioner. This service is offered to callers who have reported IIOC use and want support with working through the Get Help material. This service is not offered to callers who report additional contact offences. This anonymous and confidential service involved engaging callers in up to five calls, whereby the caller is provided with guidance and support around the online self-help modules, discuss their learning and clarify any questions about the material.

With these changes, Get Help (formerly CROGA) was launched on 13^th^ October 2015. Although these anonymous online initiatives are based on comments and feedback given by users of IIOC (Bailey et al., 2022), their impact have yet to be evaluated. Similar self-help websites exist [e.g., Troubled Desire: https://troubled-desire.com/en/ or Stoppen is mogelijk (It is possible to stop): https://stoppen-is-mogelijk.eu/], but to our knowledge, these have not yet been evaluated. A recent randomised controlled trial of the anonymous CBT intervention Prevent It with users of IIOC showed a significant reduction in IIOC use (Lätth et al., 2022).

Acceptability of an intervention is necessary for the intervention to be effective (Sekhon et al., 2017). Both deliverers of the intervention and the users of the intervention need to perceive the intervention as acceptable. Acceptability can increase the likelihood of the intervention being delivered correctly and for users to adhere to the intervention (see Sekhon et al., 2017). Sekhon and colleagues (2017) developed a multi-construct theoretical framework of acceptability (TFA): seven components are included in the TFA; *affective attitude* (how someone feels about the intervention), *burden* (the effort needed to take part in the intervention), *ethicality* (in how far the intervention aligns with someone’s values), *intervention coherence* (in how far someone understands how the intervention works), *opportunity cost* (benefits and values that need to be given up in order to engage with the intervention), *perceived effectiveness* (in how far the intervention is perceived to achieve its purposes), and *self-efficacy* (the extent to which participants believe they can perform the activities necessary for completion of the intervention). This framework has been used in previous studies to assess the acceptability of various interventions (e.g., Keyworth et al., 2022; Timm et al., 2022), including an app for young people who experienced technology assisted sexual abuse (Quayle et al., 2024).

The aim of the current research is to assess the acceptability of the online self-help website Get Help and the accompanying scheduled calls with a practitioner (called ‘call-back service’). The research question addressed in this study is: What are views and experiences of users of the self-help website Get Help relating to the acceptability of the website and scheduled calls with practitioners? Participants were interviewed about their experience with these services. Their feedback was analysed by mapping the TFA domains onto their responses.

## Method

### Study Design

This is a qualitative study with a framework approach of a sample of men who used a self-help website and calls with practitioners to address their use of IIOC. Interviews were used to gain an in-depth understanding of their experiences with the support services.

### Materials

A semi-structured interview schedule (see Appendix) was developed collaboratively by the research team and an external government stakeholder. Interviews covered participants’ process of seeking help for their offending behaviour as well as their views of the online self-help modules and call-back service.

### Procedure

Eligible participants were informed about the research at the end of their engagement with the anonymous call-back service. Participants were informed about the study and invited to participate by the practitioner they were engaging in calls with. Each participant was invited to participate in a telephone interview lasting approximately 45 minutes to one hour. Those that expressed an interest were booked in for a research telephone call with one of the researchers via a secure telephone line (where their telephone number could not be identified) at an agreed date and time. The interviews were conducted by a Forensic Psychologist and a Forensic Psychologist in Training. Due to some of the participants being undetected by law enforcement, and the importance of maintaining anonymity, participants were provided with information about the research over the telephone and given the opportunity to ask questions. Participants who remained willing to participate were read the consent form over the phone and gave consent to participate. Participants were also provided the opportunity to ask any questions prior to the interview and were debriefed at the end of the interviews. Participants were also provided with contact details of support services for any concerns arising from the research. Interview times ranged from 37 to 83 minutes, with a median of 62 minutes, and interquartile range of 27.5 minutes.

Interviews were recorded on a Homder TF-85 digital voice recorder. The interview audio recordings were transferred onto an encrypted laptop and transcribed by members of the research team. Recordings were deleted following transcription.

### Sample

All participants (*N* = 8) disclosed IIOC offending; and all participants had used both a support helpline for people concerned about their sexual thoughts or behaviour and additionally engaged in the calls with practitioners. Although a small sample, the participants represented all individuals engaging in calls with practitioners at the time of the study^1^1The Get Help website is a public website, with monthly visitors of approximately 17,000. The number of users of the additional scheduled calls is substantially lower.. Interviews took place over a six-month period in 2020.

All participants were male, aged 24 to 52 with a mean age of 38 (*SD* = 8.91). Half of the participants (*n* = 4) had been arrested for their offences related to viewing IIOC, with the remainder (*n* = 4) being undetected by law enforcement for their online offending. In terms of additional offences, two participants admitted to engaging in online sexual communication with a minor and an additional two participants admitted to engaging in sexual chat about children with other adults online. None of the participants had a previous conviction. Three participants had children, who were all under 18 years of age. Four participants were currently living with their parents, two with a partner and one in a house share with house mates (this information was not known for one participant). Three of the participants described themselves as single, four as being married and one described his status as being in a relationship.

Participants became aware of the self-help service via the police (*n* = 4), through a search engine (*n* = 3) and through media (*n* = 1).

Participants reported having been accessing IIOC for between 1 to 20 years with a mean time period of 5.5 years (*SD* = 6.69). The majority of participants (*n* = 7) reported that they had accessed IIOC over their mobile phones as this allowed easy and discreet access.

### Analysis

Transcripts were analysed using a framework approach (see e.g. Gale et al., 2013). The TFA domains (Affective attitude, Burden, Ethicality, Intervention Coherence, Opportunity Cost, Perceived Effectiveness, and Self-efficacy) were used as deductive codes to map domains of acceptability onto the data. Additional codes were inductively generated but after discussion and re-coding, these were all subsumed under the TFA domains. Coding was conducted using Taguette (Rampin & Rampin, 2021), a free and open-source computer-assisted qualitative data analysis software. Two members of the research team coded all eight transcripts and discussed the coding until full agreement was reached.

## Results

All seven TFA domains of acceptability were identified in the transcripts and are described below.

### Affective Attitude

The emotional response Get Help evoked in participants were discussed. Participants spoke about how they felt about the Get Help website before they started using it and first became aware of it: They felt that this was a place that was designed for them, where they were accepted and not judged.

“There was a palpable sense of relief, there was a sense of achievement that I had you know found this and it was through my own initiative and research and yeah, a sense of oh gosh there is actually somebody who does want to help people in my position” (Participant 1 [P1])

“It seemed to be reassuring in as much as it was fairly clear from the first page that it was a confidential helpline and that it was here to help me to try and resolve my issue with this kind of imagery” (P2)

This initial positive affective attitude changed when participants started working through the modules. They reported having experienced difficult emotions like shame when engaging with the material. Facing their own behaviours and the consequences, as well as taking responsibility for their own behaviours, was challenging. Exercises on victim empathy were perceived as particularly hard-hitting.

“I think it was that section 5, probably when I was using it I felt less confident and also I felt really ashamed (…) the exercises were telling you to put yourself in different people’s shoes and I found that difficult and upsetting and I appreciate that was the point of the exercise, so I think at that point I sort of had, I think it was a realisation of the consequences of what I was doing but it was probably the point in which I least wanted to carry on” (P2)

“I think whenever you’re told that anything is a choice and that you can change things, I find that very scary so when I encountered that in the module it was very hard to accept and deal with (…) I mean again, this is my interpretation of it but I always find that when people talk about life outside of offending or a life outside of addiction, for me I always interpret their words with quite a little bit of hostility (…) I think if it was a bit more personable and a bit more like having a conversation and then less like reading a book, it might stop – well from my perspective, it might stop defensiveness” (P6)

After working, at least partly, through Get Help, participants said they experienced a new sense of hope about the future and that they could lead a good life. They felt empowered through the work they have done.

“I certainly came away with a bit more of a renewed optimism” (P6)

Participant 6 summarised the common emotional experiences over time, from first encounter to completion of the programme:

“I think I remember at first it was a very positive reaction and I was very excited and a lot of it made sense. I think there was a few areas where I said to you it was very difficult for me to go through um and very difficult to think about the fact that you know, you were viewing these images and stuff like that but I don’t remember ever being angry about it – more – not relieved, more reassured and certainly empowered in a way um I had more understanding of my behaviour.” (P6)

The affective attitude towards Get Help changed throughout the engagement with the self-help material, starting off with feeling accepted and not judged, followed by difficult emotions while addressing their offending behaviour and underlying issues, and resulting in optimism about the future. Participants said they understood the need for the challenging exercises. To increase acceptability regarding the affective attitude, further support for users of Get Help should be implemented. This can include discussions around the impact of the self-help material and how to manage difficult emotions, guidance on how to use the material safely and advice on good self-care during and after completion of the more hard-hitting sections.

### Burden

Several issues were identified by participants that were related to the perceived effort needed to engage in the intervention. This included having difficulty finding the website in the first place, and once found, navigating the website and the modules and identifying the modules that are relevant to themselves and their situation:

“I know that like when you type into Google, I think it was the top 5 that come up it can take a bit of finding to get to the actual Get Help website. You click on the top one and it goes through to-it’s still Get Help but it’s difficult with clicks to find where you want to be (…) It’s like it’s not the first one that comes up.” (P3)

“I found it a bit tricky. I found it a bit tricky because to get to the modules I had to click on concerned about thoughts and behaviour and then Get Help and then begin the modules. So, they feel a bit hidden.” (P8)

The use of the website and the modules were hindered for some participants by dealing with technical glitches or difficulties, such as links not properly working, or the website not being optimised for use on the type of device they were using.

“I couldn’t save what I had done previously every time I had gone back on I had to kind of start again and gone through where I’d got up to.” (P3)

“I remember a few times we were having conversations and you were saying look at this exercise and I couldn’t find it on my phone because of the way of the layout on the phone didn’t mean I could see the individual chapters” (P6)

In order to work through the modules, participants invested significant amounts of their time. Their engagement usually stretched over weeks or months, with some participants regularly revisiting the work they had previously done.

“I think I used it pretty much all the time for like a few weeks” (P4)

“I used it a few times for information before the Get Help call backs and then when I was doing the Get Help call backs I was using it every time I was working on the modules so that would be on average about twice a week.” (P8)

Other burdens related to the intervention were individual difficulties reported by participants. These included having to deal with their recovery without the support of loved ones; dyslexia hindering their engagement with the content; and feeling overwhelmed with the number of modules included in the intervention:

“I think the very nature of this help that you’re seeking-this is not an alcoholic going to AA, this is not a drug user who might have a nagging mum telling them ‘Oh come on, you’ve got to get yourself clean’ or really good supportive friends (...), this is not something where you have a social or familial network behind you, this is very, very much an ‘alone thing’ that you do.” (P1)

“I mean for me, I’m quite dyslexic anyway, so (...) like reading, writing and like having that just to myself is kind of hard because I forget things a lot” (P4)

Various burdens affecting the acceptability were mentioned by participants, some of which can be mitigated. Users of Get Help can be guided more to help them navigate through the modules and identify the relevant sections for them, and further instructions can be given around how best to engage with the modules, for example, breaking down sections into smaller, more manageable segments. The design of the website needs to be optimised for different devices and the modules should be written and presented in easy-to-read language. Regarding the time invested in working through the modules, this is a burden required for meaningful engagement with the self-help modules. However, users can be instructed on the time effort needed to work through modules to shape expectations of the intervention and support users in managing their time.

### Ethicality

The Ethicality domain centres around the level to which participants felt that the Get Help modules and website aligned with their values and their views on how they want to lead their lives. Some participants felt that the programme aligned with their morals:

“I genuinely feel that I am now living the life that I wanted to live and I’m being the person that I wanted to be and it is wonderful.” (P5)

On the other hand, other participants felt that Get Help did not match their experiences and understanding of relevant factors in their problematic behaviour.

“So, it becomes impersonal. So, I mean that’s just my interpretation of it, so that can be very difficult because you can feel a little bit like, well these people don’t understand this and they don’t understand the fact that I’ve got depression and I’ve got autism and I’ve got dyslexia and there’s all these contributing factors and then I’m reading here that I can change everything and that’s not the case.” (P6)

There were mixed experiences regarding the acceptability in relation to ethicality. While some participants felt the intervention aligned with their morals and values, others felt the modules and included advice and exercises did not fit with their experiences. Further support for users with additional needs like dyslexia or autism may increase acceptability. This could include instructions of using screen readers, adjusting support to additional communication styles and needs which may be possible in scheduled calls with practitioners.

### Intervention Coherence

The contents of self-help modules and the website and how to work through them were mostly understood well. Participants said that the layout, explanations and exercises in the modules were easy to understand and to follow:

“You know when you start, you can just sort of have a look through, you realise that the website is incredibly detailed. I mean there are so many modules for you to go through, (…) it’s very very obviously a comprehensive and detailed self-help unit. (…) You know, it’s an ongoing tool, a life tool, that you can come back to again and again and again but it always seemed competent, thoroughly researched and it definitely struck the balance not being patronising, being incredibly informative, explaining why things happen and how you may have come to your pattern of behaviour.” (P1)

“No, once you’re on there it’s quite self-explanatory of where things are and what you can access and what it offers but no I think it’s quite a simple layout which it needs to be.” (P3)

On the other hand, some participants experienced some difficulty with understanding how to engage with the modules. Difficulties that participants dealt with were being unsure about the expectations or best ways of working through the modules. Also, the flow between modules was experienced as disjointed, making engagement more difficult.

“Well, I did about-I did the 17 modules and I did about 9 of them in 1 day-I’m not sure that’s how you’re supposed to do it but I wasn’t clear on that point.” (P2)

“I think it’s just it doesn’t quite flow as seamlessly as it should.” (P5)

Acceptability regarding the intervention coherence was generally high with participants who reported that they understood how the modules worked and what was required of them as a user. However, more guidance on the sequence in which to go through the modules and how to pace themselves in working through the material may be helpful for some users.

### Opportunity Costs

Several costs and obstacles that participants had to overcome or deal with in order to start or participate in the intervention were discussed. Anonymity was reported as a barrier to engaging with support. Participants were concerned that their anonymity or security could be jeopardised if they used the available support services. This concern had to be overcome to take part in the intervention.

“I was concerned about being tracked if I used the website but, in the end, I decided I was going to have to look at this anyway, so I just tried to be as anonymous as possible whilst using the website.” (P8)

Other opportunity costs were individual obstacles participants had to manage. This included having to use the internet despite this being the space where their offending took place and using this space to get help making them feel uncomfortable; or sacrificing their wish for face-to-face support to telephone support as this was the only option available.

“I mean initially I was scared I’d have to go online, and I found it a little bit strange that having an online problem, to get help was to go online-I found it a little bit strange initially.” (P3)

“I mean, face to face would always be better than over the phone but I know the situation is that some sessions (...) are expensive, so to have phone support which was free was really beneficial.” (P6)

The main opportunity cost raised was the issue around anonymity which can affect the acceptability of Get Help. The website needs to clearly communicate that users are not being tracked and that it is a safe space to seek and receive support.

### Perceived Effectiveness

According to participants, the self-help modules and calls with practitioners helped them to either stop offending or to promote their desistance from reoffending. That is, the intervention was perceived to be effective in preventing the use of IIOC in the future. Specifically, participants reported that the intervention helped them understand the triggers, antecedents and maintaining factors of their offending behaviour and how they can manage these. The calls with practitioners were perceived as creating accountability for their behaviour, motivating them to continue working on their behaviour and staying offence-free.

“Definitely, 100 percent, I mean I wouldn’t have been able to do this (address the behaviour) without [the website]- that’s a fact.” (P1)

“I’ve been, I’ve always been pretty kind of honest with you but that, there is no question that finding someone to be accountable to has been really, really important.” (P5)

“It kind of brought out a few things that you didn’t even really think about, or it made connections that I didn’t really realise were there, so it did do quite a lot about understanding my behaviour and how to deal with the choices I made and the kind of results of my choices.” (P7)

“It’s made me feel that I will be able to manage going forward because I understand the things that could lead to me going back to the offending behaviour and I know how to recognise it and what to do about them.” (P8)

In addition to helping participants stay offence-free, the intervention was also reported to have supported them in moving forward with their lives, being able to lead a more positive and fulfilling life.

“Building a Future Life, (...) I found that really, really positive because (...) it’s hard, hard things that you’re going through, so looking at yourself, looking at your behaviours, but then coming back and allowing yourself to imagine a positive future life for yourself which has been something I’ve not had all my adult life yet.” (P5)

Whilst dealing with issues of anonymity was an opportunity cost (see above), some participants also talked about how they felt the intervention was anonymous and a safe place for them to ask for and receive help. The intervention deliverers designed the intervention to be completely anonymous for participants. Where this was recognised by participants, the purpose of anonymity was achieved.

“It looks anonymous really, (...) like I read sort of the impacts- the testimonials on there, (...) it was anonymous as well so I felt like it could be trusted.” (P4)

Acceptability regarding the perceived effectiveness was high. Participants reported that the self-help modules and the scheduled calls helped them in achieving the intended aims of stopping or continuing to desist from the use of IIOC.

### Self-Efficacy

The last domain on Self-efficacy covers the participants’ level of feeling able and confident to complete the self-help modules and to address their behaviour. Several participants reported that they felt they had the necessary skills and resources to complete the modules, or that they knew what they needed to complete the modules like talking them through with the helpline.

“I needed that perhaps accountability and that ability to talk with someone about what I have written on the modules, and I think that’s the crucial point, is that this was something that I needed to speak to somebody about.” (P1)

Other participants reported that they at times did not feel able to perform the activities involved in the intervention, for example, if they were not in the right headspace to work through the modules.

“My general mood at times (...) when things have been slightly – things like the social worker and things like that might put me on edge, a little bit moody (...) so sometimes I might not have full focus.” (P7)

Participants reported mixed experiences on their level of self-efficacy in engaging with the self-help modules. Acceptability regarding self-efficacy may be further increased through guidance and instructions on when and how best to work through the modules, such as pacing themselves and taking time to work through the modules when they are in the right mental and emotional state.

## Discussion

This study aimed to explore the views and experiences of those who have been detected, as well as those who have not been detected, for IIOC offending regarding the acceptability of a self-help website to address their offending behaviour. Domains were identified from semi-structured interviews conducted with eight participants who had viewed IIOC and engaged with self-help modules online.

All seven TFA domains (Sekhon et al., 2017) were identified in the transcripts. This indicates the applicability of the framework. Acceptability before starting the intervention, while engaging in the intervention, as well as after completion of the intervention was discussed by participants, in line with the TFA’s theoretical framework of acceptability.

Overall, participants reported mixed levels of acceptability of and satisfaction with the Get Help self-help programme and calls with practitioners. Most encouragingly, the self-help modules and calls with practitioners were experienced as tools to help participants address and stop their problematic online behaviours, making positive changes to their behaviours and lifestyle (perceived effectiveness). How the intervention works was mainly understood by participants (intervention coherence) and they were largely confident that they could complete the necessary activities (self-efficacy). The modules and the calls provided a safe and non-judgemental space for participants to open up and develop a new optimism about their future (affective attitude).

The finding that participants experienced the Get Help modules and the accompanying calls with practitioners as helping them either stop offending or facilitating their capacity to desist is encouraging. The acceptability of the modules based on cognitive-behavioural therapy principles, relapse prevention, and the Good Lives Model (Ward, 2002) supports the inclusion of these approaches in self-help material for IIOC offending. These approaches are most used with people with a history of child sexual offences (Sousa et al., 2023) and have been shown to be effective in reducing reoffending amongst people convicted for sexual offences (e.g., Dowden et al., 2003; Gannon et al., 2019). The current findings suggest that these approaches may also be suitable for detected and undetected users of IIOC, because users experienced the modules as supporting them in their desistance.

Points raised by participants about difficulties and barriers in relation to the modules and the calls can be implemented by the intervention deliverers to improve the services. Considering that some modules elicited negative emotions in participants (affective attitude) while working through them, efforts should be made to manage these to maintain engagement with the intervention. The design and usability of the material should be pleasant and encouraging to facilitate engagement by making users comfortable accessing the website (Ludden et al., 2015). In addition, normalising the difficult emotions that could be experienced, within the self-help materials, could aid users with feeling more prepared for the self-help journey. Participants provided positive feedback about the accompanying calls with practitioners, and these calls may be an avenue to further prepare and help participants manage challenging emotions while working through the modules. Previous research has shown that sharing difficult emotions with others is perceived as beneficial, especially when responded to empathically (Pennebaker et al., 2001). The Get Help website could include specific encouragement for users to call and discuss their work on the self-help modules. This may support users in dealing with the emotional impact and help them achieve the other positive outcomes reported by the current participants.

Participants expressed that fear of compromising their anonymity was a barrier to engage with support services (opportunity cost). Comparatively, Levenson et al. (2017) found confidentiality issues to be a key barrier to help-seeking for those with a sexual interest in children. The confidential nature of the helpline and online self-help resources should be communicated clearly as part of the online deterrence campaign and within the messages provided on the website. Clearer explanations of how the website, and any other relevant services, allow the user to remain anonymous could aid in supporting users in engaging with the available support.

### Limitations

It should be noted that the researchers who interviewed the participants were also the professionals who had completed the calls with them. This may have led to socially desirable responses, particularly regarding the impact and their experience of the calls with the practitioners/interviewers.

A small UK-only sample was used for the current research to explore their experience with the self-help intervention. Although small sample sizes are appropriate for qualitative research, it should be noted that these findings cannot be generalised to the wider population of those who use IIOC. Additionally, participants consisted of a self-selected sample; therefore, findings may not be representative of those who have used the self-help website but did not engage in the accompanying calls or those who used the self-help website but found it unhelpful.

### Conclusion and Future Directions

Difficulties and burdens raised by participants provided valuable feedback in helping the service providers amend the self-help intervention and further increase acceptability. Efforts should be made to support users in working through emotionally challenging modules in an empathic way, reducing burdens such as technological difficulties to maintain engagement, and clearly outlining the anonymous nature of the service to facilitate first engagement with the support service. User feedback should regularly be sought to tailor the intervention to generate high acceptability amongst users. In addition to interviews, surveys on the website may be used to also reach users of the website who do not engage in calls with practitioners.

By and large, this study indicated that users of the Get Help self-help website and calls with practitioners found the service of mixed acceptability. Most notably, participants reported high perceived effectiveness of the self-help intervention. The initial feedback from participants on the acceptability of the self-help programme and the calls with practitioners warrants next steps in the evaluation of the intervention. Whether participants’ perceived effectiveness of the intervention translates to an empirically measurable reduction in IIOC use and promoted desistance from IIOC use will need to be examined in future studies. Next steps can include a full feasibility study and prospective evaluation (see Sekhon et al., 2017; Skivington et al., 2021).

## Data Availability

The data that support the findings of this study are available from the corresponding author, SW, upon reasonable request.

## Appendix – Semi-Structured Interview

### Research Semi-Structured Interview Schedule

**Problem Recognition and Seeking Help**
- **How long have/had you been accessing indecent images of children? How did this come about?** *How old were they? How did it happen? Where did they first access images?*
- **What types of devices did you use to access indecent images of children?** *Was this the same throughout their offending? Did you access on multiple devices?*
- **Why did you use those devices in particular?** *Was device type important? Or was accessing restricted to one device? If so why? Was the choice of device related to the offending?*
- **When did you first recognise that your online behaviour was a problem?** *How old were they? How long had they been offending for? Did anything trigger that recognition?*
- **When did you first consider accessing help?** *How did that thought process come about? Did anything trigger or prompt the thoughts about getting help?*
- **When did you make the decision to seek help?** *Why did they make that decision? What influenced the decision?*
- **What did you do to seek help?** *Where did they go? Did they search for help online or offline? Did they speak with anyone about their online behaviour?*
- **If you sought help online, how did you go about this?** *What search terms did they use? What were they looking for? What were they hoping to find?*
- **What help or support did you find?**

**Self-Help Website**
- **When did you first hear of the Self-Help website?** *How did this come about? Was this via the helpline? A leaflet? A google search?*
- **What was your initial reaction to the Self-Help website?** *How did they feel accessing the website for the first time? What were their first impressions of it?*
- **Did you feel comfortable using it?** *How often did they use it?*
- **How anonymous did the website feel? Did you believe that the website was anonymous?** *Did they feel concerned that it wasn’t anonymous? What was their concerns about anonymity i.e. that the website was linked to Police?*
- **What could we do to reassure you of the anonymous nature of the Self-Help website?**
- **Did you feel that the website could help you to address your behaviour and help you to move forward?** *What was their initial reaction? Did that reaction change? How confident did they feel about addressing their behaviour? Did the website influence their confidence?*
- **How could we improve the website to instil that confidence in you?** *What would it need to say? How should this be put across?*
- **When using the website did you feel encouraged to complete the modules?** *Was there any particular places where you felt your motivation dropped? Do you think more encouragement is needed? How could that be done?*
- **Did you feel that the website was there to help address your behaviour?** *Did they feel reassured that there was help? Were they confident that the content was going to be helpful? Did they feel the website was not trying to help, or trying to get another message across?*
- **Was the Self-Help site helpful?** *What did they find the most helpful? What did they find the least helpful? Do they think that there is anything we are missing? Was there anything that felt more/less relevant to their situation?*
- **Was there any information that you feel would be important for us to add?**
- **What did you think about the layout of the Self-Help site?** *Is there anything we should change?*
- **Did the Self-Help website provide you with any idea of what the future might look like?** *What did they think about this future? How did this make them feel*? Was there the sense that they can still live a good life once they had addressed the behaviour?
- **Has the Self-Help website had any impact on how you now view you online behaviour? And your ability to manage it going forward?** *Has it changed their attitudes towards their behaviour? Or their thoughts about being able to manage the behaviour?*
- **How do you think this website can best support you and others in a similar situation to yourself?**
- **You engaged in a series of call backs regarding your use of the Self-Help website. Do you think your experience of the Self-Help site would have been different if you did not do these call back?** *How would it have been different? Better or worse? What support do you think is needed when going through the website?*

Any further comments?

## Social Media Accounts

@s_wefers
